# Supplementary material for: AMARO: All Heavy-Atom Transferable Neural Network Potentials of Protein Thermodynamics
Source: J Chem Theory Comput. 2024 Nov 8;20(22):9871–8. doi: 10.1021/acs.jctc.4c01239 (PMC11603603; doi:10.1021/acs.jctc.4c01239)
Supplement: Supplementary file 1 — ct4c01239_si_001.pdf [file ct4c01239_si_001.pdf]

Supporting Information

AMARO: All Heavy-Atom Transferable Neural Network Potentials of Protein Thermodynamics

Antonio Mirarchi   Raúl P. Peláez   Guillem Simeon   Gianni De Fabritiis

**Group Type**	**Embedding Value**
C	1
CH	2
CH_2	3
CH_3	4
N	5
NH	6
NH_2	7
NH_3	8
O	9
OH	10
S	11
SH	12

Table S1: Embedding values for *noh*-beads, based on the heavy atom and the number of bonded hydrogen atoms.

Target	Target Sequence Length	Sequence Overlap (%)
Chignolin (5AWL)	10	60
Trpcage (2JOF)	20	55
Villin (2F4K)	35	51
α3D (2A3D)	73	33

Table S2: Maximum sequence similarity, σ, for fast-folding proteins compared to the mdCATH train/val/test dataset. Given a target sequence (ST) and a reference sequence (SR), then σ =(Num. Matching Residues)/|Align(ST, SR)|, where "Align" is a lexicographic alignment function and $|\cdot|$ represents the length of the alignment. Alignments were performed using Biopython's pairwise2.align.localxs function[1], with both gap open and extension penalties set to -1.

**Hyper-parameter**	**Value**
activation	Silu
aggr	add
cutoff_lower	0.0 Å
cutoff_upper	5.0 Å
embedding_dimension	128
equivariance_invariance_group	O(3)
max_num_neighbors	64
max_z	100
num_layers	1.0
num_rbf	32
precision	float32
rbf_type	expnorm

Table S3: Neural network architecture hyperparameters

Hyper-parameter	Value
optimizer	AdamW
batch size	8
distance_influence	both
early_stopping_patience	30
ema_alpha_neg_dy	1.0
ema_alpha_y	1.0
learning rate	0.0003
max_num_epochs	100
neg_dy_weight	1.0
test_interval	-1
test_size	0.1
train_size	null
trainable_rbf	false
val_size	0.05
weight_decay	null
y_weight	0.0

Table S4: Neural network training hyperparameters

CG-atom type	MAE (kcal/mol/Å)
CH0	5.56
CH1	5.02
CH2	6.05
CH3	5.44
NH0	5.17
NH1	4.27
NH2	5.30
NH3	8.10
OH0	3.53
OH1	5.09
SH0	4.79
SH1	3.54

Table S5: Mean Absolute Error (MAE) for the x, y, and z components of forces predicted by AMARO. The errors are classified by atom type across a test set of 5,000 conformations, each containing more than 150 residues, and a range of 5 temperatures. The overall MAE across all components and atom types is 4.98 kcal/mol/Å.

protein	Numb. of steps	Median length (ns)	Aggr. length (ns)
Chignolin	2.5M	10	320
Trp-Cage	3.5M	14	448
Villin	2.5M	10	320
α3D	4.5M	18	576

Table S6: Summary of the lengths of 32 MD trajectory replicas for each fast-folding protein used in the MSM analysis.

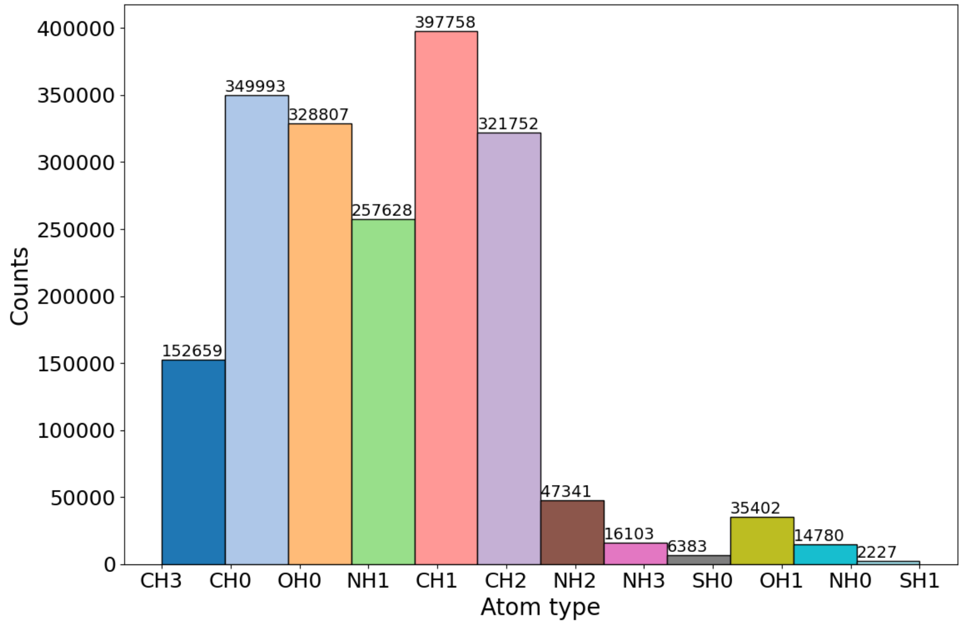

Figure S1: Distribution of coarse-grained atom types across domains in the training dataset.

**protein**	**numCluster**	**numMacordim**	**lag time (ns)**
Chignolin	350	3	0.1
Trp-Cage	350	2	0.5
Villin	250	3	0.5
α3D	600	3	0.5

Table S7: Parameters used to construct MSMs for analyzing the dynamics of different fast-folding protein trajectories simulated under AMARO.

Protein	CG-NNP			all-atom		
	Min RMSD (Å)	Mean RMSD (Å)	Macro Prob. (%)	Min RMSD (Å)	Mean RMSD (Å)	Macro Prob. (%)
Chignolin	0.16	1.11 $\pm$ 0.5	26.93	0.15	1.02 $\pm$ 0.4	57.53
Trp-Cage	0.31	3.61 $\pm$ 0.8	63.6	0.45	2.46 $\pm$ 0.82	30.1
Villin	0.6	2.7 $\pm$ 0.9	4.5	0.47	3.44 $\pm$ 1.84	69.42
α3D	2.30	3.30 $\pm$ 0.51	1.2	1.81	3.50 $\pm$ 0.75	67.89

Table S8: Minimum Average RMSD Macrostate Statistics derived from Markov State Models (MSM) built with coarse-grained simulations and all-atom molecular dynamics for fast-folding proteins. The table displays the average and minimum RMSD values (in Å) for each macrostate alongside its equilibrium probabilities, expressed as percentages (macro prob.).

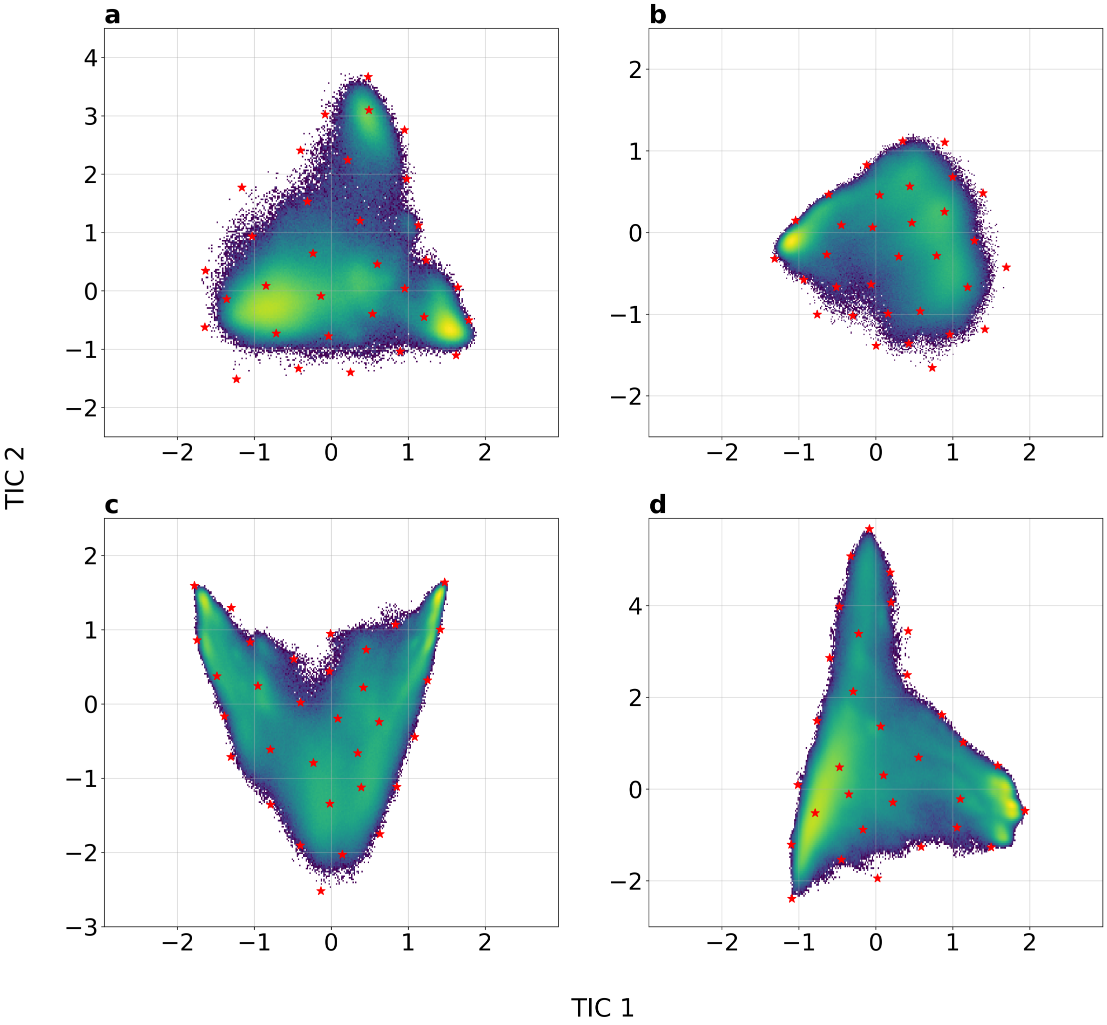

Figure S2: Initial configurations of fast-folding proteins in the test set, used to launch 32 uniformly distributed coarse-grained replica simulations across the TIC1-TIC2 space. More in details, a) Chignolin, b) Trp-cage, c) Villin, and d) α3D.

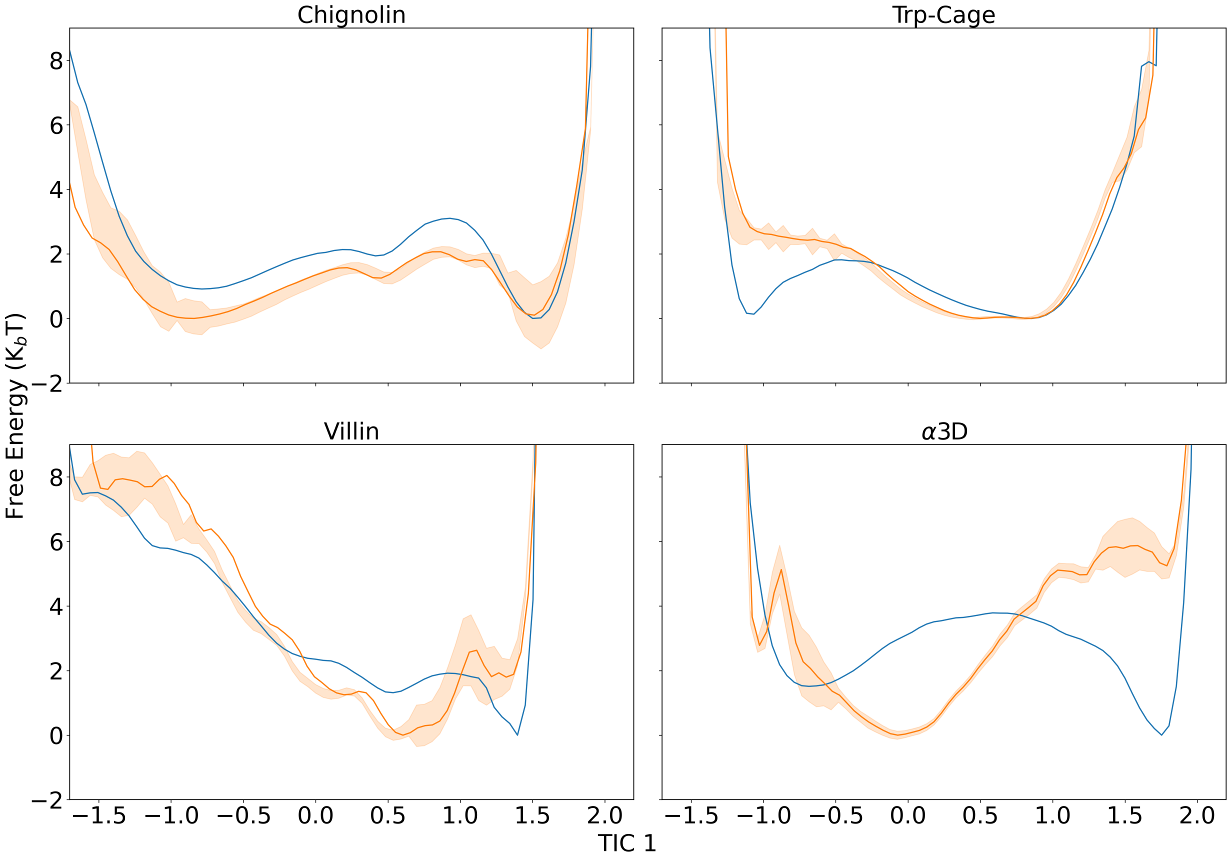

Figure S3: Free Energy comparison along the slowest TIC between NNP coarse-grained simulations (in orange) and the relative all-atom MD simulation (in blue). The standard deviation weighted over the number of replicas for AMARO is reported as shaded filling.

References

[1] Cock, P. J.; Antao, T.; Chang, J. T.; Chapman, B. A.; Cox, C. J.; Dalke, A.; Friedberg, I.; Hamelryck, T.; Kauff, F.; Wilczynski, B.; others Biopython: freely available Python tools for computational molecular biology and bioinformatics. *Bioinformatics* **2009**, *25*, 1422–1423.
